# Quantitative ultrasound (QUS) in HIV-infected patients: a reliable and low-cost technique for bone health assessment

**DOI:** 10.1186/1758-2652-13-S4-P98

**Published:** 2010-11-08

**Authors:** R Marocco, H Martini, V Belvisi, T Tieghi, R Cesareo, C Del Borgo, M Lichtner, CM Mastroianni

**Affiliations:** 1 Sapienza University, Polo Pontino, Infectious Diseases, Latina, Italy; 2 SM Goretti Hospital, Latina, Italy

## Objective

Bone demineralization is common in HIV-infected subjects. Reliable and low cost methods would be useful in order to identify and monitor bone alterations both in older and younger HIV+ patients. Recent data in normal population suggest that bone mineral quality (BMQ), assessed by a quantitative ultrasound (QUS) technique, could be an early marker of osteopenia/osteoporosis. Therefore, we investigated the usefulness of QUS in order to study bone health assessment in HIV-infected adults.

## Methods

37 HIV-infected patients and 44 HIV-negative controls, matched for sex and age, were enrolled. Bone health was measured using classical dual energy x-ray adsorptiometry (DEXA) of spine and hip and calcaneal QUS. Broadband ultrasound attenuation (BUA) and speed of sound (SOS) and quantitative ultrasound index (QUI)/stiffness index (SI) were assessed by QUS. Data were correlated with CD4+ T-cell count, HIV load, years of disease, immune activation markers (DR+CD38+CD4+ and DR+CD38+CD8+), 25OH vitamin D. Nonparametric Mann-Whitney test and Spearman correlation were used for statistical analysis.

## Results

5 patients were viremic (ARV-naïve), while the remaining subjects were virologically ARV-suppressed. In the latter group, 17 were in treatment with PI and 16 with NNRTI. The mean nadir CD4 was 665/mmc. Comparable QUS parameters were found in HIV+ subjects and controls as well as between NNRTI- and PI-based therapies. No difference was seen in patients treated with TDF. A significant decrease of QUI was found in HIV+ patients aged >48 years (p=0.012). A correlation between QUI and age in HIV+ patients was seen (p=0.047). No correlation was found between QUS and CD4 nadir, as well as between QUS and immune activation markers. No difference was observed between cases and controls for vitamin D levels which were decreased in both groups. However, a significant correlation between vitamin D level and age was found only in HIV+ population (p=0.022), other than between vitamin D and years of infection (p=0.0142). Moreover, a strong correlation between QUI and DEXA values was observed (p=0.001).

## Conclusions

QUS parameters are closely related to the structural and elastic properties of bone and they provide important information about bone quality and strength. Therefore, in HIV+ people QUS might be a simple and inexpensive technique in order to monitor bone health and identify early signs of bone damage, independently from vitamin D levels.
